# The feasibility of triggers for the integration of Standardised, Early Palliative (STEP) Care in advanced cancer: A phase II trial

**DOI:** 10.3389/fonc.2022.991843

**Published:** 2022-09-15

**Authors:** Anna Collins, Vijaya Sundararajan, Brian Le, Linda Mileshkin, Susan Hanson, Jon Emery, Jennifer Philip

**Affiliations:** ^1^ Department of Medicine, St Vincent’s Hospital, University of Melbourne, Melbourne, VIC, Australia; ^2^ Parkville Integrated Palliative Care Service, Peter MacCallum Cancer Centre & The Royal Melbourne Hospital, Melbourne, VIC, Australia; ^3^ Department of Medical Oncology, Peter MacCallum Cancer Centre, Melbourne, VIC, Australia; ^4^ Cancer Australia, Sydney, NSW, Australia; ^5^ Department of General Practice, University of Melbourne, Melbourne, VIC, Australia; ^6^ Palliative Care Service, St Vincent’s Hospital Melbourne, Melbourne, VIC, Australia

**Keywords:** early palliative care, outpatient palliative care, cancer, personalized palliative care, clinical trial, phase II

## Abstract

**Background:**

While multiple clinical trials have demonstrated benefits of early palliative care for people with cancer, access to these services is frequently very late if at all. Establishing evidence-based, disease-specific ‘triggers’ or times for the routine integration of early palliative care may address this evidence-practice gap.

**Aim:**

To test the feasibility of using defined triggers for the integration of standardised, early palliative (STEP) care across three advanced cancers.

**Method:**

Phase II, multi-site, open-label, parallel-arm, randomised trial of usual best practice cancer care +/- STEP Care conducted in four metropolitan tertiary cancer services in Melbourne, Australia in patients with advanced breast, prostate and brain cancer. The primary outcome was the feasibility of using triggers for times of integration of STEP Care, defined as enrolment of at least 30 patients per cancer in 24 months. Triggers were based on hospital admission with metastatic disease (for breast and prostate cancer), or development of disease recurrence (for brain tumour cohort). A mixed method study design was employed to understand issues of feasibility and acceptability underpinning trigger points.

**Results:**

The triggers underpinning times for the integration of STEP care were shown to be feasible for brain but not breast or prostate cancers, with enrolment of 49, 6 and 10 patients across the three disease groups respectively. The varied feasibility across these cancer groups suggested some important characteristics of triggers which may aid their utility in future work.

**Conclusions:**

Achieving the implementation of early palliative care as a standardized component of quality care for all oncology patients will require further attention to defining triggers. Triggers which are 1) linked to objective points within the illness course (not dependent on recognition by individual clinicians), 2) Identifiable and visible (heralded through established service-level activities) and 3) Not reliant upon additional screening measures may enhance their feasibility.

## Introduction

Patients with advanced cancer suffer numerous distressing physical symptoms, psychological morbidity and unmet information and psychosocial needs (1–4). Despite assigning high priority to symptom relief, open communication and collaborative decision making (5), such needs are frequently not recognised nor managed in routine oncology care (6, 7).. Addressing these needs are core tasks of palliative care, and increasingly meta-analyses demonstrate the benefits of early palliative care for patients, including improved symptom management, quality-of-life and care satisfaction; reduced rates of hospitalization and emergency department presentations, and for family carers, improved quality-of-life and care satisfaction (8–13).

Despite benefits and recommendations from peak professional bodies (ASCO, ESMO) (14–16), in practice ‘early’ palliative care referrals are not routine and access to palliative care frequently occurs very late in the illness course (17). Our earlier work demonstrated only 59% of decedents with metastatic non-small cell lung, small cell lung, prostate and breast cancers in Victoria, Australia received a palliative approach to care, a median of 27 days prior to death (17). A repeat of these analyses (almost 10 years on) for decedents from cancer in 2018 revealed 67% had a palliative care referral, but at a later time, median 20 days before death (18). Equivalent data on cancer decedents from the United Kingdom (19) and other international jurisdictions demonstrated palliative care referral 53 days and 18.9 days prior to death respectively (20). As such, there remains a significant evidence-practice gap associated with the implementation of early palliative care in routine cancer care.

Barriers to palliative care referral have been identified, including: concerns about difficulty of referral, fear of destroying patient hope associated with perceptions of palliative care (21) and uncertainty over the ‘best time’ to refer (22). The literature has variably defined ‘early’, including to mean at least 3-4 months prior to death to confer benefits (8), within 3 months of advanced cancer diagnosis for patients with a life expectancy of 1 year or less (23), and ideally engagement with palliative care spanning 6-18 months before death (24).

An approach which seeks to standardise the timing of ‘early’ palliative care referral would do much to overcome such barriers, including through increasing patient acceptance of referrals, as it represents a ‘routine’ care pathway (25). Similarly, standardisation would reduce variations and inequities in access to care. Such a standardised time of introduction should be based on evidence and be tailored to the disease characteristics and likely history, allowing for consideration of balancing the potential for maximal outcome benefit versus managing the volume of early consultations and resourcing implications (23, 24). Yet to date, few studies have explored the role of systematic triggers for timely palliative care referral (26–29).

We previously examined population level hospital admission datasets to map health care use by patients with high grade glioma (HGG) and metastatic breast, prostate as well as lung cancers (17, 30–32). This work demonstrated potential disease-specific transition points in the illness course which heralded subsequent poor prognosis (defined as less than 6 months) and subsequent increased health service utilisation (17). These ‘transition points’ or ‘triggers’ represented times for the integration of early palliative care as part routine clinical practice when we recommended that palliative care should be routinely introduced, if not already in place, to maximise patient and carer benefit (33). These triggers for palliative care are linked with electronic health records or usual systems of clinical care, may prompt clinicians and in this way, serve to augment clinician-based decision making (24). However, there is a clear need for the testing of such cancer specific time points as triggers for referral to palliative care occurring as ‘standard quality care’ (17).

Responding to this gap, we undertook a randomised, phase 2 feasibility trial of a standardised outpatient model of ‘early’ palliative care [Standardised Early Palliative Care: STEP Care] for advanced cancer patients and their family carers, with referrals occurring at the defined disease-specific, evidence-based trigger points. The trial sought to test the feasibility and preliminary efficacy of using defined triggers for the the integration of standardised, early palliative (STEP) care across three advanced cancers. This paper reports on the feasibility of triggers.

## Methods

### Study setting

The trial was undertaken at four metropolitan tertiary cancer services in Melbourne, Victoria, each with active inpatient and outpatient palliative care consultation services. Central multi-site ethical approval was provided by the Human Research Ethics Committee at St Vincent’s Hospital Melbourne [HREC 179/16], and the trial registered with the Australian and New Zealand Clinical Trial Registry [ACTRN12617000534381]. Funding was provided by the Victorian Cancer Agency [Grant number: HSR15022] and the St Vincent’s Hospital Foundation (private philanthropic donation).

### Patient and public involvement

The trial had patient and public involvement embedded within the research team (SH), and additionally through the guidance of an advisory group comprising community contributors who met regularly with the research team (quarterly meetings) across the life of the trial. This group had a significant role in shaping the following areas: grant application, review of patient consent forms and plain language summary, review of language to introduce the study, input into selection of research outcomes and qualitative question guides, trouble-shooting recruitment, and grounding interpretation of study results.

### Primary endpoint

The primary outcome was the feasibility of using triggers for times of integration of STEP Care, with a view to proceeding to a definitive Phase 3 randomised trial, which would evaluate effectiveness of STEP Care (compared to usual best practice cancer care) for patients with advanced breast or prostate cancer or high grade glioma (HGG). The specific feasibility endpoint was defined as enrolment of at least 30 patients in each disease cohort (total n=90) in 24 months, at which time those cancers not meeting feasibility cut off were ceased. Secondary aims to examine the preliminary efficacy of STEP Care on patient- and carer- reported outcomes, including quality of life, mood, symptoms, illness understanding, and overall survival will be reported elsewhere. Consistent with the exploratory study aims, the feasibility endpoint was determined by the authors primarily balancing pragmatic considerations around the available study timeframe. It was consistent with other phase II studies of this nature (34) and also considered the minimum sample required to determine a preliminary estimate of effect size for secondary patient-reported outcomes which would be the subject of a future phase III definitive trial.

### Design

We conducted a phase 2, multi-site, open-label, parallel-arm, Randomized Controlled Trial (RCT) of usual Best Practice Cancer Care +/- STEP Care according. This RCT development aligned with the Medical Research Council (MRC) Framework for the development and testing of Complex Interventions (35, 36) which prioritises phased, sequential, intervention development leading towards implementation (35, 36). The nature and timing of the triggered early palliative care was thus underpinned by our exploratory data resulting from Phase 1 qualitative (22, 37–39) and health service use studies (17, 30, 31, 40) which defined transition points or triggers for the integration of early palliative care.

### Triggers for standardised early palliative (STEP) care

The triggers for STEP Care as defined for this feasibility trial included (**Table 1**): for prostate- first multiday admission where patient had any metastatic disease; for breast- first multiday admission where patient had metastatic disease including at least one visceral site; for brain- any hospital presentation with first recurrence of HGG (determined by radiological or surgical evidence). Given our earlier state-wide population cohort studies of cancer decedents found first palliative care occurred a median of <30 days prior to death (17, 18), these triggers were selected to offer an objective time for systematic identification of a cohort likely to benefit from palliative care earlier in the disease trajectory.

**Table 1:** Trigger definitions.

Characteristics and identification of cases meeting the trigger		
Prostate cancer	Presence of metastatic disease AND Multi-day hospital admission.	Presence of advanced disease ANDChange in care requirementANDHeralded *via* electronic health record
Breast cancer	Presence of visceral metastatic disease (metastases involving organs other than bone only) ANDMulti-day hospital admission.	Presence of advanced diseaseAND Change in care requirement ANDHeralded *via* electronic health record
High grade glioma	First recurrence of primary HGG where pathological or clinical diagnosis is Glioblastoma/ WHO grade IV disease; ORFirst diagnosis of primary HGG and no cancer specific treatment being prescribed. ANDHospital presentation (inpatient or outpatient)	Illness based (e.g. new point in illness course*)ANDHeralded in usual systems of clinical care (illness point anchored to key treatment decision discussed at multidisciplinary cancer meetings)

*time of new complication or disease progression determined by radiological and surgical evidence.

The point of hospitalisation with the disease characteristics outlined was selected because it was: not reliant upon individual clinician judgement of prognosis or of the person’s needs; common to most patients with these cancer illnesses; and could be identified within the electronic health record. An anticipated life expectancy of between 6 and 24 months has been advocated as appropriate for patient inclusion in early palliative care (24, 41). These points of hospitalization were previously found in our population studies to have a median survival of approximately 6 months (42), thus balancing the imperatives for early palliative care input against common service concerns about capacity to respond (41) and relevant to the variable and not infrequently long metastatic illness course experienced particularly by the breast and prostate cohorts.

### Participants

Participants included adult patients with advanced breast, prostate and brain cancers as identified by the defined triggers (**Table 1**), and in attendance at the included hospital sites at this time. Further eligibility requirements included the ability to provide informed consent, to comply with study procedures, and an ability to understand written and spoken English. Exclusion criteria for patients included those less than 18 years, those previously seen by hospital consultancy palliative care services within the previous 12 months or presenting with needs required urgent palliative care review, or those who were more than 30 days following the identified cancer-specific trigger. Patients meeting the eligibility criteria who were identified by a mechanism other than the route specified (**Table 1**) could be included in the study, however none were referred in this way.

### Study Procedures

#### Recruitment and consent

Consecutive eligible inpatients and outpatients from participating cancer treatment centres were approached for potential study inclusion by research staff. At patient identification, clinical teams were asked to confirm eligibility, permission was sought from the patient to provide information about the study, with those willing to proceed completing a study consent form. Information on eligibility along with reasons for refusal to participate were recorded.

#### Randomisation

Patient-level randomisation was centralised and coordinated by an independent Trial Coordinator. The randomisation schedule involved 1:1 allocation and used the minimisation method to ensure a balanced distribution between groups with respect to the patient’s tumour type and hospital site.

#### Usual care: Standard Best Practice Cancer Care

All patients received usual oncological care through their health care providers, including systemic therapy, radiotherapy, surgery or other treatments deemed appropriate. In addition, those patients randomised to usual care were able to be referred to palliative care services at any time at the treating clinician’s discretion.

#### Intervention: STEP Care plus Standard Best Practice Cancer Care

Those patients randomised to the intervention arm received STEP Care in addition to Standard Best Practice Cancer Care. STEP Care consisted of, at minimum, monthly Palliative Care consultations for at least 3 months. These consultations were primarily delivered in the outpatient setting. All STEP Care consultations were conducted by a Palliative Care Physician or Specialist Nurse and involved a series of activities (**Table 2**) which were documented according to a framework adapted from the PC-NAT-PD (43).

**Table 2:** Key components of STEP Care intervention.

Identification of patients for eligibility at defined trigger in the illness course. Initial hospital based palliative care consultation, addressing: Review of underlying disease management Screening for symptom distress Screening for psychological distress Review of informal social supports Review of formal community supports, including local community palliative care Providing information Advance care planning discussions Involvement of family carer, including enquiry of concerns, needs for information Regular follow up, at minimum monthly for minimum of 3 months. Case conference with the general practitioner within 28 days, addressing Current and anticipated problems. Recommended management and therapies Designation of responsibility for different aspects of care.

#### Data collection

Demographic, clinical, and treatment data were collected from patient medical records. Mixed method study data were collected to assess the feasibility and acceptability of the triggers as prompting referral to the standardized early palliative care intervention. Measures of feasibility were assessed according to the number of eligible participants identified, consented and completing the study. Acceptability of the STEP Care intervention was assessed according to the number of withdrawals from the study, the completeness of delivery and timing of STEP care consultations for those assigned to the intervention arm, and the development of any adverse events. In addition, semi structured qualitative data with providing perspectives of purposively sampled participating oncology and palliative care clinicians, was supplemented to explore issues of feasibility and acceptability associated with using triggers for the integration of early palliative care.

### Analyses

Feasibility outcomes were summarised using descriptive statistics, including frequency counts and percentages (categorical variables), and mean/standard deviation or median/interquartile range (continuous variables) as appropriate. Qualitative data aligned to the primary outcome of feasibility and acceptability was subjected to a thematic analysis (44) to supplement the basic descriptive analyses consistent with the study aims.

## Results

### Participant characteristics

#### Patients

Of 513 patients identified as meeting the cancer-specific trigger point (141 brain, 118 prostate, 254 breast), 406 were not eligible to approach for study participation (58 brain, 106 prostate, 242 breast), most commonly owing to already being linked into palliative care (n=183, 45%), or presenting with needs requiring immediate referral to palliative care (n=71, 18%), or cognitive impairment (n=42, 10%) (**Table 3**). Of the 107 patients identified as eligible, 42 (39%) declined study participation, mostly citing they were not interested at this time (23, 55%) as opposed to high levels of distress (n=3, 7%), or the time commitment involved (n=2, 5%). The remaining 65 (61%) participants were consented for study participation and underwent random assignment.

**Table 3:** Feasibility and acceptability data.

**Domain**	**Measure**	**HGG**	**Prostate**	**Breast**	**Total**
Feasibility data	**Identified as ineligible**	141	118	254	513
	Reason for ineligibility				
	Cognitive impairment	21	7	14	42
	More than 30 days since trigger	6	0	2	8
	Already receiving palliative care	27	55	101	183
	Needs imminent palliative care	24	15	32	71
	Language other than English	7	11	13	31
	Receiving treatment elsewhere/regional	23	–	–	23
	Other (eg. on another clinical trial, advice of treating clinician)	33	30	92	155
	**Identified as eligible**	N=83	N=12	N=12	107
	Declined participation	34	2	6	42
	Reason for declining				
	too distressed	2	0	1	3
	not interested	19	1	3	23
	time commitment	2	0	0	2
	other	11	1	2	14
	Consented to participation	49 (58%)	10 83%)	6 (50%)	65 (61%)
	Median (IQR) time from trigger to death or study completion (months)	7.1 (4, 14)	33.7 (10, 39)	32.2 (8, 32)	
Acceptability of STEP Care to patients and carers	Assigned to STEP Care study arm	24	5	5	34
	Completion of first STEP Care consultation within 14 days of consent	19 (86%)	4 (80%)	4 (80%)	27 (79%)
	Days from consent to first STEP interaction	10 (0, 12)	2 (1, 4)	2 (0, 4)	5 (0,12)
	Number of consultations per patient	3 (2, 4)	4 (2, 4)	1 (1, 2)	3 (2, 4)
	Received at least 3 STEP Care consultations	18 (75%)	3 (60%)	1 (20%)	22 (65%)
	Number of consultations per patient within first 3 months	3 (2, 3)	3 (2, 3)	1 (1, 2)	2.5 (2, 3)
	Number of withdrawals from Trial (STEP Care) intervention	2 (8%)	0	1 (20%)	3 (8.8%)
	Number of adverse events arising from Trial (STEP Care intervention)	0	0	0	0

#### Participating clinicians

Interview and focus group data was obtained from oncology and palliative care clinicians (n=19) who were directly or peripherally involved in the STEP care trial as a member of the treating teams involved in the care of included breast, prostate or brain cancer patients. This included perspectives from palliative care nurses (n=3) and consultants (n=6), and oncology nurses (n=3) and consultants (n=7).

### Feasibility of triggers

The triggers underpinning times for the integration of standardised, early palliative care (STEP care) were shown to be feasible for brain, but not breast or prostate cancers, with total enrolment of 49, 6 and 10 patients across the three disease groups respectively. The breast and prostate groups were determined not feasible and ceased at the pre-specified 24 month timeframe, with recruitment for the brain cohort (then n=38) continuing through to 36 months.

#### Timing of identified triggers

The cancer specific triggers used in this trial appeared to be ‘*too late’* for the breast and prostate groups, with high rates of these participants identified already having a previous palliative care referral (breast: 101/254, 40%; prostate: 55/118, 47%) as compared to the brain group (27/141, 20%), and additional breast (32/254, 13%) and prostate (15/118, 13%) cancer patients identified as having urgent palliative care needs. These data suggest earlier involvement may have been helpful.

“If there’s been an admission in the setting of metastatic disease that can often mean that there are symptoms and they’re not doing so well at home and (we) get the palliative care team involved” (Oncology consultant)

“Many of the patients (breast, prostate) have been seen by palliative care already” (Palliative care nurse)

**Figure 1:**
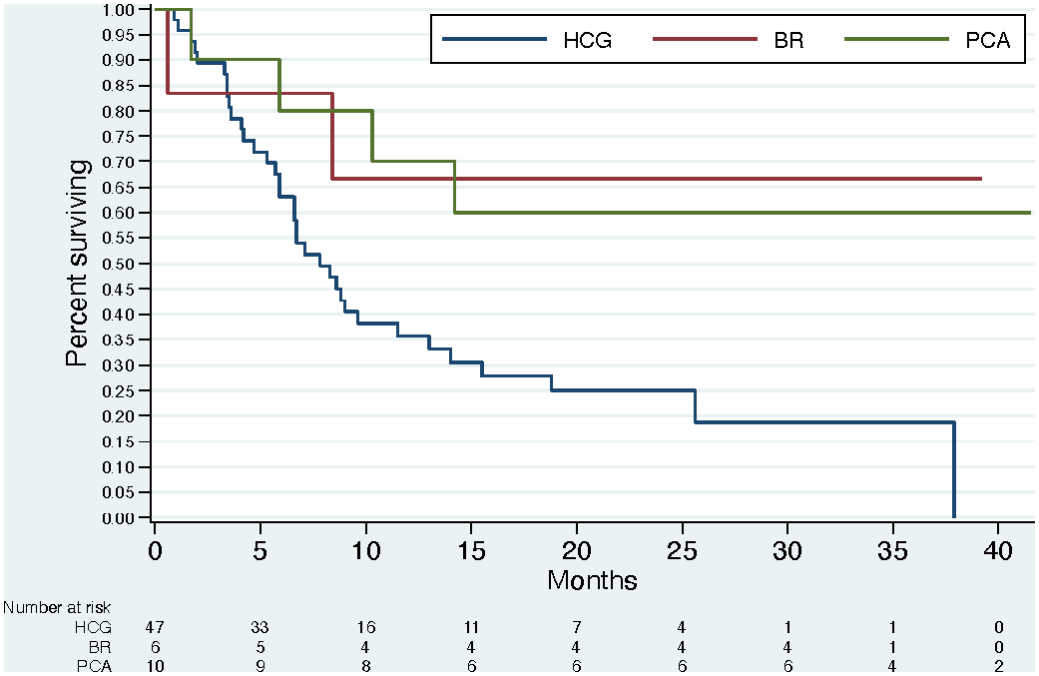
Overall Survival by Cancer Type.

On the other hand, the median overall survival of the cohort from the identified trigger until death or censored at study completion was 9 months (**Figure 1**). The median follow-up time from the trigger was: for brain 7.1 (4.1, 14.1) months, for breast 32.15 (8.4, 32.4) months, and for prostate 33.65 (10.3, 39.4) months. This suggests the triggers were aligned with a period where a person is likely to benefit from palliative care, and highlights the resourcing challenge in groups such as breast and prostate where a person may experience palliative care needs over a long metastatic illness course.

#### Characteristics of a feasible trigger

The feasible trigger associated with care of the brain cancer cohort was illness based (e.g. at time of new progressive disease or a new complication of the illness) and heralded in usual clinical systems of care (anchored to key treatment decision discussed at multidisciplinary cancer meetings). The ‘not feasible’ triggers of breast and prostate cancer care were at a time of advanced illness (though not necessarily a new development of progressive disease or complication), when care requirements changed and required screening of the electronic health medical records to identify patients.

Qualitative data from clinicians revealed that electronic medical records within the included hospital settings were not yet established for *real time* prompting of eligible patients. This was largely because relevant data such as the cancer diagnosis that may be uncovered within the admission and recorded in the patient’s electronic health record was only ‘coded’ by hospital administrative teams following the patient discharge. This meant that administrative teams could not generate an automatic list of ‘eligible’ patients meeting the identified trigger in real time, which reduced the feasibility for breast and prostate triggers since it therefore required usual care teams to additionally screen inpatients for eligibility. This was compared to the brain patient cohort, where the trigger was anchored to a new illness development which prompted discussion in usual clinical care systems – specifically the multidisciplinary cancer team meeting. For brain patients, no additional surveillance over and above usual care processes was required to identify people meeting eligibility.

“Having an easy mechanism of referral is really important … I think there’s particular patients when we’ve got some big life decisions to discuss at our M.D.M, that would be a good time to bring in palliative care” (Surgical Oncology – Urology)

“I think it provides a standardised pathway that you can offer to patients, and an easy access pathway … and it keeps it at the front of your mind.” (Oncology nurse)

### Acceptability of a trigger to STEP Care Intervention

Of the 65 participants, 34 were assigned to receive the STEP Care Intervention (24 brain, 5 breast, 5 prostate). Of these, 27 (79%) completed the first consultation within 14 days as per protocol, a median (IQR) of 5 days (0, 12) following identification and consent (**Table 3**), suggesting the responsiveness of the palliative care teams who were able to facilitate an initial review within the planned timeframe. Most patients (22, 65%) received a ‘minimum dose’ of 3 (monthly) consultations as prescribed, with a median (IQR) of 3 (2, 4) consultations per patient across the study period. These data suggest that the timing of the trigger was broadly acceptable to patients who continued to attend appointments. Of note, there were 3 patients (9%) who withdrew from the STEP Care intervention due to increasing illness burden, and no adverse events recorded.

#### Standardisation of practices

The triggers also appeared to be acceptable to clinicians who described standardization of practices around referral to palliative care referral as reassuring to both themselves and the patients.

“It (the trigger) gives permission to refer people and it is normalised under the medical pathway … I think. the formality … gives it a much more medical procedural thing rather than an esoteric, nebulous sort of thing … by having the defined (trigger) points” (Oncology consultant)

“Before. it was difficult because … I felt I needed … some problem to be able to put in that referral. Whereas having a trigger allows us to be able to much more fluidly, you know, send through that referral.” (Oncology nurse)

“The key you know, (having) flags that teams can recognise as a point for a referral as opposed to … where it could be a bit more subjective. These clear kind of delineated flags for a referral … certainly gets our foot in the door with a lot of patients earlier” (Palliative care consultant)

#### Triggers as reducing communication barriers

Having a trigger also meant that conversations around referral to palliative care were easier.

“I think something like this for a junior clinician nurse, it gives them something tangible that they can open the discussion with” (Oncology consultant)

“There’s none of that having to break through the barrier of, you know, referring to pall care. It’s just an automatic thing so there’s no barrier to break because it happens all the time anyway.” (Oncology nurse)

#### Trigger and intervention set the scene for longer term care

For most patients, clinicians perceived the 3 consultations delivered at time of the trigger was adequate to introduce the role of early pc, put some key plans in place, help with family discussions, and facilitate relationships so that subsequent contact could be initiated by the patient or their carer if and when the need arises.

“…It’s really good to have that concurrent pathway where we can link patients in from an early point,…As things progress it makes things much easier when you get further down the line as well, in terms of having them already linked in, knowing what services are available and making that transition.” (Oncology Consultant)

“some (patients) at those earlier stages … may have a significant survival trajectory still but have other potential symptoms or things that could be managed in the interim period of time … so they’re not getting to the end stage before being referred” (Palliative Care Consultant)

Similarly for palliative care clinicians, having triggers was perceived as a means to build relationships between palliative care and referring teams that enabled the longer term care of patients to be met. In this way, the triggers were seen as providing patients with a universal opportunity to be linked with palliative care.

“Although there’s lots of rhetoric about taking a population-based approach to palliative care, when you are constrained by resources, you retreat and do what you just have to do to manage, don’t you. So, I think this has been really positive in helping us look at these specific groups, and it’s increased out dialogue with our referrers.”(Palliative Care Consultant)

#### Limitations of triggers

As noted, the defined triggers for prostate or breast cancer patients were not useful since many patients were already linked to palliative care services, or already had high supportive care needs identified which had prompted earlier referral.

“I think just maybe having a look at the (trigger) points and just seeing umm how, if there’s certain groups that are coming in … too late. And then just revising those.” (Palliative Care Consultant)

Other staff highlighted that while having a trigger was useful for some patients, the circumstances of other patients necessitated the need for flexibility around timing of palliative care referral.

“I tend to tailor it per patient rather than having an automatic criteria for which I would refer someone because I just think everyone’s very individual.” (Surgical oncology)

Similarly, triggers were sometimes seen as interfering with practices of a staged approach to the introduction of palliative care or the providers ‘clinical intuition’ regarding the right time.

“I don’t think right now is the best time for me to … refer to palliative care. But, you know, as weeks go on and they settle in, you develop—we develop, as nurses and clinicians there, the best way of knowing what is the right time to introduce it.” (Cancer nurse)

## Discussion

Identifying the cohort of people who will benefit from palliative care and enacting this access in a timely manner requires new approaches in service delivery. This trial tested the feasibility of novel, evidence-based, cancer-specific, illness-based triggers for the integration of standardized early palliative care across three advanced cancer groups. The triggers as defined were shown feasible by our endpoint for the brain but not prostate or breast cancer groups. Achieving the implementation of early palliative care as a standardized component of quality care for all oncology patients will require attention to further defining triggers which can help reduce variation and enhance the equity of care. In this trial the successful trigger was characterized by being 1) linked to objective points within the illness course at a new development in the illness (thus, not dependent on recognition by individual clinicians), 2) Identifiable and visible (heralded through established systems of clinical care or service-level activities) and 3) Not reliant upon additional screening measures. While these are early data in the field, these characteristics are likely to be important to inform the development of feasible triggers going forward.

In this study, and others (45) we have sought through exploring triggers to test a universal approach to identifying the group of people who may benefit from palliative care. A handful of other single-centre studies have similarly examined models of ‘triggered palliative care consultation’, often also initiated on criteria involving hospitalisation, and these have reported variable outcomes (26, 27, 29). Adelson and colleagues used a hybrid of automatic criteria relating to health service use (prior hospital within 30 days; or > 7 bed days) and active symptoms for prompting palliative care referral, resulting in a two-fold increase in rates of consultation and a significant reduction of hospital re-admission (26). Rocque and colleagues demonstrated improved illness understanding following implementation of triggered palliative care for all hospitalised cancer patients with metastases, but this resulted in a minimal impact upon patient-reported symptoms, hospice utilisation, and cost of care (29). DiMartino and colleagues reported triggered palliative care for hospitalized solid tumour and gynecologic patients increased uptake, but this did not result in earlier timing of consultations (27).

Our approach to standardizing early palliative care differed in that it sought to test the feasibility of cancer specific triggers to initiate a prescribed palliative care intervention, which was then delivered in outpatient settings. The triggers, defined upon pre-identified health service parameters, differed for different cancers, and thus meant our results also reflected some nuance in the understanding of different cancer types and the feasibility of the respective triggers. In this way we have begun to define those characteristics of a successful trigger and also of those not likely to be successful. In this trial, a successful trigger was linked to a clear, new development in the illness, was identifiable and heralded in usual service systems, and did not rely on additional screening. Since the characteristics of services differ, local factors will necessarily inform the implementation of such a trigger into routine practice. The views of the referrers as to the acceptability of the trigger as point of referral to palliative care will be essential, with a successful trigger one that reflects and is adapted to local service conditions and agreed upon by referrers.

In the context of this clinical trial, with necessarily tight eligibility parameters, the triggers enacted for prostate and breast cancer were shown to be not feasible, or ‘too late’. This was largely reflecting the high number of people already receiving, or needing imminent palliative care at the identified trigger, thus rendering them ineligible in the clinical trial context. Despite this, it was interesting that our survival data on the participants in these cohorts, albeit small numbers, was broadly consistent with the literature recommending palliative care input for those with a life expectancy of 6-24 months (24, 41). Going forward in clinical practice and outside of a trial setting, this may suggest that these trigger points as outlined are not unreasonable as a ‘minimum standard’ to prompt the initiation of palliative care if not already in place. Alternatively, these triggers could be adapted to earlier in the disease course, such as at the time of second line treatment. In this case, these triggers could be linked to identification *via* the systems whereby care is reviewed such as in the multi-disciplinary cancer meeting. Preliminary pilot testing, as undertaken here, would first be required to establish feasibility.

An alternative approach to using triggers, is to instead focus palliative care referral prompted by needs, with those identified as having greater or complex needs receiving specialist palliative care (46, 47). Such an approach seeks to target the limited resources of palliative care upon those who may benefit most, and is based in a population-centred model. The concept of ‘complexity’ at the centre of this approach however is not well defined (48). Furthermore, in order for referral of those with complex needs to occur, an assessment of needs by referring clinicians must take place. Such an assessment is frequently not part of their usual consultation, is not built into usual workflows and would constitute an additional task in an already busy consultation. As such it may be overlooked. Even when such needs are assessed, acting upon these does not occur routinely for many patients (7, 49).

Hui et al. (24) have attempted to bring this discussion of triggers and needs together in a service innovation which seeks to apply routine systematic screening, an established defined set of referral criteria which, if reached, triggers a referral to palliative care for appropriate patients. In addition an adequately staffed outpatient specialist palliative care service is available to respond to these referrals (24). In this way standardisation of practice is achieved with attendant equity of access for patients, but focused on those with greatest needs who may most benefit. The resources required for the systematic screening and implementation in this model will not however, be available in a number of centres.

Our focus on using triggers which may be built into usual care systems offers an approach which also will standardise the time of referral and address issues of equity of access. The opportunity to automate these triggers based in electronic systems associated with electronic medical records means fewer resources are required to standardise identification of the patient cohort. An electronic prompt to clinicians could serve as a reminder, reducing clinical uncertainty and reinforcing the service expectations (24). Clinicians, so prompted, could consider their response which may include consideration of activities of palliative care such as review of symptom burden, or discussion of goals and preferences, or it may include a referral to specialist palliative care. A system using electronic prompts needs to be as accompanied by clearly communicated but not overly prescriptive guidance, thus reducing uncertainty whilst not reducing physician agency (50). An effective trigger-prompt system would be one where clinicians are reminded of palliative care benefits and retain the decision making about how and when those are best enacted.

There are limitations to this trial that require mention, including a focus on those patients who were cared for in large cancer centres (where neuro-oncology units exist) and who may not be representative of all cancer patients. Similarly those people who did not speak English were excluded - a group which constitutes up to 21% of the Australian population (51). Furthermore our study was around the feasibility of using triggers for a trial of early palliative care, not simply referral to palliative care for all comers. As such, the eligibility criteria to enter the trial were likely to rule out some patients that may otherwise have welcomed (or benefited from) palliative care referral. This includes some participants excluded based on other clinical trial participation. Given the increasing potential for many patients to be accessing clinical trials of novel systemic therapies moving forward, future early palliative care trials may need to carefully consider this parameter, which will likely substantially reduce the available sample who may otherwise benefit from early palliative care. Nonetheless, by structuring the feasibility of triggers as time for referral within a trial, we were able to measure outcomes in a standardized formal manner including delivery and acceptability. We recognise that there are many parameters which impact upon feasibility and acceptability outcomes and our trial necessarily chooses selected measures likely not capturing all of these attendant influences.

We contend that key to the implementation of early, timely palliative care into clinical care is the development of novel ways of identifying the cohort of people who will benefit. The use of triggers offers an approach which provides standardization of the cohort identification and therefore will reduce variation and enhance equity of access to early palliative care. Characteristics of a successful trigger are that it is linked to a clear, new development in the illness, is identifiable and heralded in usual service systems and does not rely on additional screening. Future research focused upon linking these triggers to electronic clinical prompt systems offers interesting ways forward. The need to tailor the triggers and attendant responses to local conditions will be core to successful implementation endeavours.

## Data availability statement

The raw data supporting the conclusions of this article will be made available by the authors, without undue reservation.

## Ethics statement

The trial involving human participants was reviewed and approved by St. Vincent’s Hospital Melbourne HREC. All participants provided their written informed consent to participate in this study.

## Author contributions

JP was the lead investigator and obtained the study funding. JP & AC were responsible for the study conduct, had access to the data, and co-authored the first draft. All authors contributed to the study protocol, interpretation and, contributed to the manuscript.

## Funding

Funding was provided by the Victorian Cancer Agency [Grant number: HSR15022] and the St. Vincent’s Hospital Foundation (private philanthropic donation).

## Conflict of interest

The authors declare that the research was conducted in the absence of any commercial or financial relationships that could be construed as a potential conflict of interest.

## Publisher’s note

All claims expressed in this article are solely those of the authors and do not necessarily represent those of their affiliated organizations, or those of the publisher, the editors and the reviewers. Any product that may be evaluated in this article, or claim that may be made by its manufacturer, is not guaranteed or endorsed by the publisher.
